# A national survey on violence and discrimination among people with disabilities

**DOI:** 10.1186/s12889-018-5277-0

**Published:** 2018-03-15

**Authors:** Jesper Dammeyer, Madeleine Chapman

**Affiliations:** 1 Department of Psychology, University of Copenhagen, Øster Farimagsgade 2a, 1353 Copenhagen K, Denmark; 2 Department of Psychology, University of Westminster, London, England

**Keywords:** Discrimination, Mental disability, Physical disability, Physical violence, Sexual violence, Domestic violence

## Abstract

**Background:**

The aim of the study was to quantify levels of violence and discrimination among people with disabilities and analyze the effects of gender and the type and degree of disability.

**Methods:**

The study analyzed data on self-reported violence and discrimination from a Danish national survey of 18,019 citizens, of whom 4519 reported a physical disability and 1398 reported a mental disability.

**Results:**

Individuals with disabilities reported significantly higher levels of violence than those without. Specifically, individuals reporting a mental disability reported higher levels of violence and discrimination. Significant gender differences were found with regard to type of violence: while men with disabilities were more likely to report physical violence, women with disabilities were more likely to report major sexual violence, humiliation and discrimination. Neither severity nor visibility of disability was found to be a significant factor for risk of violence.

**Conclusions:**

This large-scale study lends support to existing research showing that people with disabilities are at greater risk of violence than people without disabilities. Further, the study found that people with mental disabilities were significantly more likely to report all types of violence and discrimination than those with physical disabilities. The findings also show that gender is significant in explaining the type of violence experienced and the experience of discrimination.

## Background

### Understanding disability

The World Health Organization (WHO) uses the term disability to refer to the physical or mental impairment of everyday functionality due to congenital conditions, injury or disease [1]. While this study follows the WHO conceptualization of disability as comprising impairment of everyday functionality, it is grounded in the understanding that people are disabled by the intersecting effects of impairment and social attitudes and barriers [2]. Further, the study is guided by the understanding that many mental health issues such as depression and anxiety may be caused by a combination of factors, including environmental and experiential factors. In this study, the term disability encompasses these understandings and is used to refer to a range of mental as well as physical health issues, including developmental disorders such as autism spectrum disorder and mood disorders such as depression. The key objective of this study is to explore the relationship between different types of disability and the experience of violence and discrimination.

### Violence and discrimination among people with disabilities

Interpersonal physical and sexual violence contribute significantly to the global burden of physical and mental health problems, substance abuse, and early mortality [3]. As the World Report on Disability [4] highlights, people with disabilities are at greater risk of violence than those without disabilities. Due to ageing populations and the increasing global burden of disease and injury, the prevalence of disability worldwide – now estimated at 15% of adults – is predicted to rise [4], further underlining the importance of more research on the experience of violence among people with disabilities.

There are few studies that compare risks of violence among different disability groups within a large sample and none that compare risks of discrimination.

One large-scale study that highlights disability as a risk factor for violence is Khalifeh et al. [5] which analyzed data gathered from 44,398 adults through the British Crime Survey and estimated the risks of experiencing past-year violence. After adjusting for socio-demographic and other confounders, the study found that people with disabilities were at increased risk of experiencing violence compared to people without disability, with the risk greatest for those with mental disabilities (with relative odds ratio of 3.0). In their systematic review and meta-analysis of research on violence and disability, Hughes et al. [6] reported similarly that adults with disabilities were at a significantly higher risk of violence compared with non-disabled adults. They also found that the experience of past-year violence was highest (at 24%) among individuals with mental disabilities.

Even though a higher prevalence of violence among those with mental disabilities has been reported, there are no clear findings on the type of violence experienced nor the effects of other factors such as the specific type and severity of disability. However, gender has been reported to be an important factor. For example, Khalifeh et al. [7] found that women with chronic mental illness reported higher levels of intimate partner violence than men with chronic mental illness (the figures for reported past-year violence were 21% for women and 10% for men).

Disability-related discrimination is an issue of interest in legal and social science literature on disability [8]. There are some studies that draw attention to the implications of discrimination for well-being, for example highlighting links between underemployment, perceived discrimination, and negative well-being [9]. However, there are no large-scale studies on disability-related discrimination nor studies that address different forms of discrimination and examine the effects of various factors such as type, severity and visibility of disability. Some disabilities are clearly visible to others, thereby exposing individuals more to the risk of disability-related discrimination, but others may not be readily apparent. Such factors need to be addressed in research to better understand the operation of discrimination as distinct from violence.

### Objectives

Existing studies give a picture of the greater risks of violence faced by people with disabilities. However, there is an identified need [6] for more large-scale studies and, specifically, studies examining interactions between different forms of violence and possible risk factors. Khalifeh et al. [5] urge in particular further research on which subgroups of people with mental disabilities are at greatest risk of violence. Further, there is a need for large-scale research using detailed measures on disability-related discrimination. Thus, the objectives of this study are to provide an overview of violence and discrimination among people with disabilities through analysis of data from a national survey; and to compare the prevalence of different forms of violence and discrimination with respect to gender and the type, severity, and visibility of disability.

## Methods

### Sampling and participants

Data for this study were drawn from the Survey of Health, Impairment, and Living Conditions in Denmark, collected by The Danish National Centre for Social Research in 2012/2013 [10]. Using personal identification numbers, Statistics Denmark generated a random selection of 32,810 citizens aged from 16 to 65 years old. Selectees were sent an invitation with instructions about how to complete the questionnaire online. If they did not respond, they were offered a phone interview. Of the selectees, 18,957 (57.8%) responded, with 15,292 (81%) completing the questionnaire online and 3665 (19%) participating in a phone interview. Consent to participate was obtained for the Survey of Health, Impairment, and Living Conditions in Denmark.

The mean age of the participants was 43.3 (*SD* = 14.13). Of the total number of participants, 46.8% were men. Regarding education, 13% had completed five years or more of tertiary education, 38% had completed some years (less than five) of tertiary education, 29% had completed secondary education, and 20% had completed primary education.

### Questionnaire and measurements

#### Physical and mental disabilities

The main exposures were physical disability and mental disability. In the questionnaire, participants were asked if they had “a long-term physical health problem or disability” and/or “one or more mental disorders”. They were then asked to categorize their most serious physical and/or mental disability. The study grouped the response categories for physical disabilities as follows: (1) Motor and movement disorders; (2) Blindness and vision loss, despite use of glasses or contact lenses; (3) Deafness and impaired hearing, despite use of hearing aids or cochlear implants; speech and language difficulties; dyslexia; (4) Skin diseases; (5) Allergies and breathing difficulties; (6) Chronic conditions and progressive diseases; (7) Other health problem or disability. The questionnaire provided examples for the categories, such as cerebral palsy for motor and movement disorders. The study grouped the response categories for mental disabilities as follows: (1) Mental disorder caused by alcohol or substance use; (2) Schizophrenia and psychosis; (3) Mood disorders including depression and bipolar disorder; (4) Stress, phobias, anxiety, obsessive-compulsive disorder (OCD), post-traumatic stress disorder (PTSD); (5) Personality disorders, (6) Autism spectrum disorders; (7) Attention deficit hyperactive disorder (ADHD) or similar disorder; (8) Eating disorders; (9) Other mental disorders.

#### Severity and visibility of disability

The study also used measures of the severity and visibility of disability. In the questionnaire, participants were asked if their main physical and/or mental disability was “minor or major”. They were also asked: “Would a stranger recognize within five minutes that you have a disability/health problem/mental disorder?” The response categories for the latter were coded by this study as “always” and “sometimes/never”.

#### Violence

The outcomes were forms of violence and discrimination. For violence, the outcomes were separate forms of violence in the past 12 months, including physical violence, sexual violence, and non-physical violence. For non-physical violence, the question was: “In the past year, has someone: (1) Threatened you with violence (2) Humiliated, degraded or ridiculed you, or constantly criticized you; (3) Prevented you from accessing your money or bank account, blocked your bank card, or forced you to pay a sum of money or act as guarantor?” For physical violence, the question was: “In the past year, has someone: (1) Shaken you, pushed you or pulled your hair; (2) Hit or kicked you?” The study refers to (1) and (2) as “minor” and “major” physical violence respectively. A question on violence done to others was also included in this study, as follows: “Have you shaken, pushed, hit or kicked another person within the last year?” For sexual violence, the question was: “In the past year, has someone forced you to: (1) Kiss or hug; (2) Have sexual intercourse or engage in other sexual acts?” The study refers to (1) as “minor sexual violence” and (2) as “major sexual violence”.

#### Discrimination

In the survey, the following definition of discrimination was provided: “Discrimination occurs when people are unfairly treated because they are perceived as different from others.” In the questionnaire, participants were asked: “Do you feel that you are discriminated against because of your disability?” Participants were then asked who discriminates against them (naming only the most important). Response categories were grouped into two for this study, as follows: (1) Employment and education (e.g. work managers, personnel at college/university, colleagues); (2) Services (e.g. staff in public administration, health-care professionals, home carers, and support staff, staff in shops, cinemas, buses, trains etc.).

### Data analysis

Descriptive analysis of the frequency of violence and discrimination was carried out with respect to gender, type of disability, and severity and visibility of disability. Chi-square tests were completed to compare differences for outcomes on the basis of mental and physical disability, specific types of mental and physical disability, severity and visibility of disability, and gender. Further, logistic regressions models were built, with types of violence and discrimination as dependent variables, and gender, age, level of educational achievement, physical disability and mental disability as independent variables.

## Results

### Violence

As reported in Table 1, individuals with disabilities were significantly more likely to report all types of violence committed against them than those without disabilities. For example, whereas 3.2% (*n* = 403) of those without disabilities reported major physical violence in the last year, the figures were 3.8% (*n* = 173) for those with physical disabilities and 6.7% (*n* = 93) for those with mental disabilities. The regression models (see Table 6) showed that having a physical and mental disability was significant for all types of violence.. For example, the odds ratio for reporting major sexual violence was 4.30 for participants with a mental disability compared to participants without a mental disability.

**Table 1:** Frequency of self-reported past-year violence and discrimination among participants with and without physical and mental disability

Disability	Threatened physical yes % (n)	Humiliated yes % (n)	Financial yes % (n)	Physical minor yes % (n)	Physical major yes % (n)	Sexual minor yes % (n)	Sexual major yes % (n)	Violence to others yes % (n)	Discrimination employment and education yes % (n)	Discrimination services yes % (n)
No disability (*n* = 12,707)	7.1(896)	11.5(1460)	0.6(74)	4.8(608)	3.2(403)	2.1 (261)	0.7(89)	2.8(355)	–	–
Physical disability (*n* = 4519)	8.8(398)*	16.5(744)**	1.1(50)*	5.7(258)*	3.8(173)*	2.7(121)*	1.3(60)*	2.9(131)ns	2.8(126)	2.5(114)
Mental disability (*n* = 1398)	13.3(186)**	27.5(384)**	2.8(39)**	10.1(141)**	6.7(93)**	6.7(93)**	3.9(55)**	6.2(86)**	4.2(59)	3.8(53)
Both mental and physical disability (*n* = 635)	12.9(82)**	27.7(176)**	2.8(18)**	10.1(64)**	6.9(44)**	5.8(37)**	3.6(23)**	5.4(34)**	4.3(27)	6.1(39)
Mental compared to physical disability	**	**	**	**	**	**	**	**	*	*

Note: Chi square statistics for comparisons with participants with “no disability”, *p < .05, **p < .01, ns = not significant

People with mental disabilities were significantly more likely to report all types of violence than those with physical disabilities. This included violence done to others: 6.2% (*n* = 86) of those with mental disabilities reported this, compared to 2.9% (*n* = 131) of those with physical disabilities.

#### Severity and visibility of disability

No significant differences were found for violence with respect to severity of disability and visibility of disability (see Table 2).

**Table 2:** Frequency of self-reported past-year violence and discrimination with regard to severity and visibility of physical and mental disability

Severity and visibility of disability	Threatened physical yes % (n)	Humiliated yes % (n)	Financial yes % (n)	Physical minor yes % (n)	Physical major yes % (n)	Sexual minor yes % (n)	Sexual major yes % (n)	Violence to others yes % (n)	Discrimination employment and education yes % (n)	Discrimination services yes % (n)
Minor physical (*n* = 3087)	8.5(262)	15.8(487)	1.0(30)	5.6(173)	3.9(119)	2.5(76)	1.2(37)	3.1(95)	2.0(61)	0.9(27)
Major physical (*n* = 1414)	9.3(132)ns	17.9(253)ns	1.4(20)ns	5.9(83)ns	3.6(51)ns	3.1(44)ns	1.6(23)ns	2.5(35)ns	4.6(65)**	6.1(86)**
Minor mental (*n* = 998)	12.0(120)	26.2(261)	2.5(25)	9.2(92)	6.3(63)	6.3(63)	3.5(35)	6.5(65)	3.5(35)	2.6(26)
Major mental (*n* = 384)	15.9(61)ns	30.8(118)ns	3.1(12)ns	12.0(46)ns	7.0(27)ns	7.0(27)ns	4.7(18)ns	5.2(20)ns	6.0(23)ns	6.8(26)*
Stranger ever/sometimes (*n* = 4985)	9.3 (465)	18.1(900)	1.3(64)	6.4(319)	4.2(211)	3.4(167)	1.7(87)	3.5(172)	2.9(144)	2.0(100)
Stranger always (*n* = 289)	12.1(35)ns	17.3(50)ns	2.1(6)ns	5.2(15)ns	3.5(10)ns	3.1(9)ns	1.4(4)ns	3.8(11)ns	4.5(13)ns	9.7(28)**

Note: Chi square statistics comparisons, *p < .05, **p < .01, ns = not significant

#### Gender and violence

Differences were significant for all categories of violence for men and women with mental disability as compared, respectively, with men and women without disability (Table 3). There were no significant differences in reporting of physical and sexual violence between men with physical disabilities and men without. For women with either physical or mental disabilities, reporting of all categories of violence was significantly higher than for women without disabilities, except for major physical violence for women with physical disability. For example, 0.8% (*n* = 55) of women without disabilities reported major sexual violence compared with 1.7% (*n* = 42) of women with physical disabilities and 5.0% (*n* = 45) of women with mental disabilities.

**Table 3:** Frequency of self-reported past-year violence and discrimination with regard to gender among participants with physical and mental disability

Gender	Threatened physical yes % (n)	Humiliated yes % (n)	Financial yes % (n)	Physical minor yes % (n)	Physical major yes % (n)	Sexual minor yes % (n)	Sexual major yes % (n)	Violence to others yes % (n)	Discrimination employment and education yes % (n)	Discrimination services yes % (n)
Men no disability (*n* = 6159)	9.2(566)	10.6(655)	0.7(41)	5.8(356)	4.0(245)	2.1(130)	0.6(34)	4.0(247)	–	–
Women no disability (*n* = 6548)	5.0(330)	12.3(805)	0.5(33)	3.8(252)	2.4(158)	2.0(131)	0.8(55)	1.6(108)		
Compared to men no disability	**	*	ns	**	**	ns	ns	**	–	–
Men physical disability (*n* = 1989)	11.7(233)	15.4(307)	1.3(25)	6.6(131)	4.7(93)	2.5(50)	0.9(18)	3.9(78)	2.0(40)	1.8(36)
Compared to men no disability	*	**	*	ns	ns	ns	ns	ns	–	–
Women physical disability (*n* = 2530)	6.5(165)	17.3(437)	1.0(25)	5.0(127)	3.2(80)	2.8(71)	1.7(42)	2.1(53)	3.4(86)	3.1(78)
Compared to women no disability	*	**	*	*	ns	*	**	ns	–	–
Compared to men physical disability	**	ns	ns	*	*	ns	*	**	*	*
Men mental disability (*n* = 493)	15.2(75)	24.2(119)	2.6(13)	10.6(52)	6.3(31)	4.7(23)	2.0(10)	6.7(33)	2.4(12)	2.8(14)
Compared to men no disability	**	**	**	**	*	**	**	*	–	–
Women mental disability (*n* = 905)	12.3 (111)	29.3(265)	2.9(26)	9.8(89)	6.9(62)	7.7(70)	5.0(45)	5.9(53)	5.2(47)	4.3(39)
Compared to women no disability	**	**	**	**	**	*	**	**	–	–
Compared to men mental disability	ns	*	ns	ns	ns	*	*	ns	*	ns

Note: Chi square statistics comparisons, *p < .05, **p < .01, ns = not significant

Comparing men and women with mental and physical disabilities, respectively, women were significantly more likely to report major sexual violence than men. Men with physical disabilities were significantly more likely than women with physical disabilities to report physical violence, the threat of violence, and violence against others. Comparing women and men with mental disabilities, there were no significant differences in reports of physical violence, the threat of violence, and violence against others. These findings were reflected in the regression models (see Table 6) which showed that gender was significant for all kinds of violence except for financial violence and minor sexual violence. The odds for men were higher than for women for physical violence, being threatened and violence against others. The odds for women were higher for major sexual violence and being humiliated compared to men.

#### Disability type and violence

With regard to type of physical disability, those with a motor disability reported higher levels of being threatened and lower levels of being humiliated than people with all other kinds of physical disability. Compared to people with all other kinds of physical disability, people with visual impairment reported higher levels of being humiliated, financial violence, major physical violence, and both minor and major sexual violence. Finally, those with allergies reported higher levels of being threatened, being humiliated, and minor and major physical violence than people with all other kinds of physical disability (see Table 4).

**Table 4:** Frequency of self-reported past-year violence and discrimination with regard to type of physical disability

Physical disability type	Threatened physical yes % (n)	Humiliated yes % (n)	Financial yes % (n)	Physical minor yes % (n)	Physical major yes % (n)	Sexual minor yes % (n)	Sexual major yes % (n)	Violence to others yes % (n)	Discrimination employment and education yes % (n)	Discrimination services yes % (n)
Motor (*n* = 2222)	9.4 (208)*	15.8(350)*	0.9(20)ns	6.1(135)ns	3.4(76)ns	2.3(50)ns	1.0(22)ns	2.9(65)ns	3.2(70)**	2.6(58)**
Vision (*n* = 67)	11.9(8)ns	22.4(15)*	4.5(3)*	7.5(5)ns	9.0(6)*	9.0(6)*	4.5(3)*	6.0(4)ns	3.0(2)ns	4.5(3)*
Hearing (*n* = 89)	4.5(4)ns	15.7(14)ns	1.1(1)ns	1.1(1)ns	0.0(0)ns	3.4(3)ns	1.1(1)ns	3.4(3)ns	0.0(0)ns	4.5(4)*
Skin (*n* = 87)	4.6(4)ns	20.7(18)ns	2.3(2)ns	4.6(4)ns	2.3(2)ns	3.4(3)ns	1.1(1)ns	1.1(1)ns	1.1(1)ns	2.3(2)ns
Allergy (*n* = 426)	12.9(55)**	19.0(81)*	0.5(2)ns	9.9(42)**	8.0(34)**	2.8(12)ns	1.2(5)ns	4.5(19)ns	1.4(6)ns	1.2(5)ns
Chronic (*n* = 987)	6.9(68)ns	13.9(137)ns	1.0(10)ns	3.7(37)*	2.6(26)ns	1.8(18)ns	1.2(12)ns	2.1(21)ns	2.3(23)**	2.1(21)**
Other (*n* = 579)	8.3(48)ns	20.4(118)**	2.1(12)*	5.7(33)ns	4.7(27)ns	4.8(28)*	2.8(16)**	2.8(16)ns	4.0(23)**	3.5(20)**

Note: Chi square statistics comparisons with “participants with all other physical disabilities”, *p < .05, **p < .01, ns = not significant

With regard to mental disability, the overall finding was that people with personality disorders, ADHD, autism spectrum disorder, and schizophrenia/psychosis reported significantly higher levels of violence than people with other kinds of mental disability (Table 5). People with stress and mood disorders reported significantly lower levels of violence than people with other kinds of mental disability.

**Table 5:** Frequency of self-reported past-year violence and discrimination with regard to type of mental disability

Mental disability type	Threatened physical yes % (n)	Humiliated yes % (n)	Financial yes % (n)	Physical minor yes % (n)	Physical major yes % (n)	Sexual minor yes % (n)	Sexual major yes % (n)	Violence to others yes % (n)	Discrimination employment and education yes % (n)	Discrimination services yes % (n)
Alcohol and drugs (*n* = 15)	26.7(4)*	40.0(6)*	6.7(1)ns	13.3(2)ns	6.7(1)ns	6.7(1)ns	6.7(1)ns	0.0(0)ns	0.0(0)ns	6.7(1)ns
Schizophrenia and psychosis (*n* = 47)	19.1(9)*	29.8(14)*	0.0(0)ns	12.8(6)*	8.5(4)ns	8.5(4)*	6.4(3)*	6.4(3)ns	6.4(3)*	6.4(3)*
Mood (*n* = 542)	11.1(60)*	24.9(135)**	1.7(9)*	9.2(50)**	5.7(31)*	5.4(29)**	3.7(20)**	5.9(32)**	3.7(20)**	2.8(15)**
Stress (*n* = 517)	11.8(61)*	29.2(151)**	3.9(20)**	8.7(45)*	5.6(29)*	5.6(29)**	3.1(16)**	5.2(27)*	4.8(25)**	4.3(22)**
Personality (*n* = 29)	34.5(10)**	31.0(9)*	6.9(2)*	24.1(7)*	20.7(6)**	20.7(6)**	6.9(2)*	20.7(6)**	3.4(1)ns	10.3(3)*
Autism (*n* = 23)	40.9(9)**	36.4(8)*	4.5(1)ns	27.3(6)*	13.6(3)*	9.1(2)ns	9.1(2)*	4.5(1)ns	8.7(2)*	13.0(3)*
ADHD (*n* = 51)	26.0(13)**	40.0(20)**	4.0(2)ns	18.0(9)*	14.0(7)*	16.0(8)**	6.0(3)*	12.0(6)*	5.9(3)*	3.9(2)ns
Eating (*n* = 24)	8.3(2)ns	20.8(5)ns	0.0(0)ns	16.7(4)*	12.5(3)ns	12.5(3)*	0.0(0)ns	0.0(0)ns	4.2(1)ns	4.2(1)ns
Other (*n* = 144)	11.8 (17)ns	25.0(36)**	2.8(4)*	7.6(11)ns	6.3(9)ns	7.6(11)*	5.6(8)**	7.6(11)*	2.8(4)*	2.1(3)ns

Note: Chi square statistics comparisons with “participants with all other mental disabilities”, *p < .05, **p < .01, ns = not significant

### Discrimination

People with mental disabilities were significantly more likely to report discrimination of both categories than those with physical disabilities (Table 1). Severity of physical disability was significant with respect to discrimination: those with major physical disabilities reported higher levels of discrimination in both categories than those with minor physical disabilities (Table 2). Those with major mental disabilities reported significantly higher levels of discrimination in services than those with minor mental disabilities. Finally, those with visible disabilities reported significantly higher levels of discrimination in services than those without.

With regard to gender, women with physical disabilities were significantly more likely than men with physical disabilities to report discrimination of both categories (Table 3). Women with mental disabilities were significantly more likely than men with mental disabilities to report discrimination in employment and education. In line with this, the regressions models showed that gender was significant for both types of discrimination (see Table 6).

**Table 6:** Logistic regression models for predicting violence and discrimination

	Threatened physical		Humiliated		Financial		Physical minor		Physical major	
Predictors	OR(95% CI)	*p*-value	OR(95% CI)	*p*-value	OR(95% CI)	*p*-value	OR(95% CI)	*p*-value	OR(95% CI)	*p*-value
Gender (female = 1)	1.89(1.69–2.13)	<.001	.85(.78–.93)	<.001	1.23(.88–1.72)	.220	1.43(1.24–1.64)	<.001	1.59(1.35–1.88)	<.001
Age (one year decrease)	1.05(1.05–1.05)	<.001	1.04(1.03–1.04)	<.001	1.03(1.02–1.05)	<.001	1.07(1.06–1.07)	<.001	1.07(1.06–1.07)	<.001
Level of educational achievement (one unit decrease)	1.04(1.01–1.06)	.013	1.02(1.00–1.04)	.113	1.06(.97–1.15)	.204	1.08(1.04–1.12)	<.001	1.05(1.01–1.10)	.025
Physical disability (no = 1)	1.58(1.39–1.81)	<.001	1.58(1.43–1.74)	<.001	1.61(1.12–2.32)	.010	1.61(1.37–1.89)	<.001	1.63(1.35–1.98)	<.001
Mental disability (no = 1)	1.90(1.60–2.27)	<.001	2.41(2.11–2.75)	<.001	3.95(2.69–5.81)	<.001	2.07(1.70–2.53)	<.001	2.03(1.60–2.58)	<.001
	Sexual minor		Sexual major		Violence to other		Discrimination employment and education		Discrimination services	
Predictors	OR(95% CI)	*p*-value	OR(95% CI)	*p*-value	OR(95% CI)	*p*-value	OR(95% CI)	*p*-value	OR(95% CI)	*p*-value
Gender (female = 1)	.95(.78–1.15)	.584	.55(.40–.76)	<.001	2.23(1.85–2.68)	<.001	.46(.33–.65)	<.001	.48(.33–.70)	<.001
Age (one year decrease)	1.06(1.05–1.07)	<.001	1.05(1.04–1.07)	<.001	1.08(1.07–1.09)	<.001	.99(.98–1.00)	.046	.99(.98–1.00)	.234
Level of educational achievement (one unit decrease)	1.09(1.04–1.15)	.001	1.10(1.01–1.18)	.021	1.07(1.02–1.12)	.006	1.02(.95–1.10)	.563	1.28(1.18–1.39)	<.001
Physical disability (no = 1)	1.41(1.12–1.77)	.003	1.59(1.14–2.21)	.007	1.42(1.14–1.76)	.002	–	–	–	–
Mental disability (no = 1)	3.01(2.35–3.86)	<.001	4.30(3.07–6.02)	<.001	2.44(1.89–3.14)	<.001	–	–	–	–

Note: OR = Odds Ratios, CI = Confidence Intervals; Level of educational achievement (1 = primary school to 8 = 5 years or more tertiary education); Multicollinearity check: Variance Inflation Factors were all between 1.01–1.03

Regarding type of disability, those with a motor disability, autism spectrum disorder, or schizophrenia/psychosis were significantly more likely than those with other types of physical or mental disabilities, respectively, to report discrimination of both categories (Tables 4 and 5). Those with a personality disorder were significantly more likely to report discrimination in services and those with ADHD were significantly more likely to report discrimination in employment and education. By contrast, those with a stress or mood disorder were significantly less likely to report discrimination of both categories.

## Discussion

The findings here lend support to existing research showing that individuals with disabilities are at increased risk of violence [5, 6]. By analyzing data on various kinds of violence, the study indicates that the increased risk is associated with all the forms of violence measured: physical, sexual, and non-physical. The finding that participants with mental disability reported significantly higher levels of all categories of violence and discrimination than those with physical disability are in line with other studies showing the particular vulnerabilities of people with mental disabilities [5, 6]. However, more focused studies are required to explore more precisely the nature of the relationship between mental disability and violence and discrimination (see Limitations).

The study’s findings in relation to gender both build upon and nuance previous research on disability and violence [7] and highlight the need for an intersectional approach to disability studies. Whereas men with disability reported more physical violence, women with disability reported more humiliation, discrimination and major sexual violence. For example, 5% (*n* = 45) of women with mental disabilities in this study reported past-year major sexual violence. This compares to the estimated EU-wide figure of 5% of women who have been raped since the age of 15 [11]. The finding of this study underlines an urgent need for more research to address the nature of the relationship between mental disabilities among women and the experience of sexual violence.

Neither the severity nor visibility of disability were significant in explaining the risk of violence. While perhaps surprising, this is in line with previous studies which have found that the degree of physical impairment does not always predict life outcomes [12]. However, this study found that degree of physical disability was significant for both categories of discrimination. Further research with respect to who commits violence and discrimination could shed light on risk factors, both within and outside the home.

The findings from this study suggest that women with disabilities are at increased risk of discrimination than men with disabilities. and further that those with mental disabilities are at greater risk of discrimination than those with physical disabilities. This study’s findings from a large-scale survey provide an overview of risk that can connect piecemeal studies on discrimination and disability, such as studies highlighting cases of workplace discrimination among those with mental health problems [13], and further underline the need for an intersectional understanding of risk.

Regarding findings with respect to disability type, attention is directed to the high levels of violence and discrimination reported by those with personality disorders, schizophrenia/psychosis, ADHD, and autism spectrum disorder. On discrimination specifically, the findings direct attention to motor disorders in addition to the above. However, these findings are offered with caution because of small group numbers.

### Limitations

The first main limitation of this study might be a participation bias. The survey was designed for the general population and may have excluded those with severe cognitive impairment or communication difficulties. Further, this study did not include people above 65 years of age, thereby excluding a group of people among whom the prevalence of physical disabilities is high [14]. However, this had an advantage, which was the default exclusion of much age-related discrimination and violence.

The second main limitation of this study was that the data on disabilities were based on self-report rather than validated diagnoses of disability. This might have led to either under- or over-reporting of disability. However, the survey’s detailed level of questioning about type of disorder and its use of diagnostic terms were designed to promote specificity and reliability of reporting.

There may also be a self-report bias with respect to violence and discrimination. This is likely to be in the direction of under-reporting. First, as Hughes et al. [6] observe, the past-year criterion for violence likely results in conservative estimates as “many more will have suffered violence more than 12 months previously” (p.1627). Second, there may have been a disclosure bias with respect to domestic violence. As Khalifeh et al. [5] observe, it may be particularly difficult for people with disabilities to report domestic violence because of dependency on perpetrators and fear of institutionalization. It should also be noted here that there is some controversy about the ability of those with serious mental disorders to report traumatic events. However, research suggests the reliability of self-report among this group. For example, Goodman et al. [15] concluded from their research that the occurrence and severity of violence are reliably reported by people with serious mental health illness.

Another limitation of this study was that the cross-sectional design did not enable clear identification of whether disability or violence occurred first. However, this issue is largely mitigated by the past-year criterion for occurrence of violence, a study condition that Hughes et al. [6] applied for inclusion in their systematic review, and by the survey’s qualification of physical disabilities as “long-term”. As the survey did not provide this qualification for mental disabilities, the implications of findings with respect to mental disabilities are discussed in the study with more caution. For some kind of disabilities, bi-directional and compounding effects of violence and disability might exist [16, 17]. For example, Khalifeh et al. [5] found that people with disabilities were at greater risk of psychological health problems following violence than non-disabled people. This study included participants’ reports on doing violence to others and found that significantly higher levels were reported by participants with personality disorder and ADHD. The experience of multiple victimization and the compounding effects of negative experience are topics that need further attention.

One further limitation concerns the inclusion of data relating to those with both physical and mental disabilities (*n* = 635) in the data for physical and mental disability groups respectively. While this potentially resulted in the over- or under-statement of rates of violence and discrimination, the decision was made to be inclusive of all those reporting physical and mental disabilities by not stripping out co-morbidity.

Finally, the number of cases within some of the disability groups was low and therefore any conclusions for these groups should be taken with caution.

### Strengths of study

The strengths of this study were the large national sample and detailed survey data about the type and severity of disability and different forms of violence and discrimination. This enabled robust comparative analysis of the prevalence and risk of violence and discrimination.

## Conclusions

Understanding the extent of violence against vulnerable groups is the first step in starting to combat it [6]. While this study’s design constrains conclusions, the findings here call attention to the significantly higher levels of violence reported by people with disabilities and particularly those with mental disabilities. Clinicians and other health professionals as well as service providers and employers should be cognizant of these patterns of vulnerability and the intersections with gender and other risk factors.

Further to increased awareness and screening, this study highlights the need for coordinated assessment of causes of violence and policies on prevention as well as treatment and support. Specifically, the high level of sexual violence among women reporting mental disabilities demands further inquiry and action. Further to this, the consistently high levels of reported threats of violence, humiliation, and financial abuse suggest the need for agencies to identify and tackle non-physical and domestic violence. This requires attunement to the factors contributing to the vulnerabilities of people with disabilities, including dependency on personal care, social isolation, and communication difficulties [6, 18]. Specialist services to support people with disabilities facing domestic violence are required but seldom in place [5].

Finally, the findings on disability-related discrimination call for greater awareness of this issue and its social costs in terms of employment, education, and service provision. Discrimination should be approached with an understanding of its “institutionalized” nature which, to paraphrase the U.K. Macpherson Report on racism [19], can be “seen or detected in processes, attitudes, and behaviour which amount to discrimination through unwitting prejudice, ignorance, thoughtlessness, and stereotyping which disadvantage people with disabilities.” The findings here hopefully provide some grounding for future research and action on discrimination that addresses multiple factors and the apparently increased risk for those with mental disabilities and women with disabilities.

## Abbreviations

ADHD: Attention deficit hyperactive disorder
OCD: Obsessive-compulsive disorder
PTSD: Post-traumatic stress disorder
WHO: World Health Organization
